# Quantification of Mitral Valve Regurgitation in Cavalier King Charles Spaniels and Chihuahuas Using Radius of Proximal Isovelocity Surface Area

**DOI:** 10.3390/ani14192805

**Published:** 2024-09-28

**Authors:** Jørgen Koch, Inge V. Engeland, Jakob L. Willesen, Anders S. Schrøder, Caroline H. Gleerup, Maiken B. T. Bach

**Affiliations:** 1 Department of Clinical Veterinary Sciences, Faculty of Health and Medical Sciences, University of Copenhagen, Dyrlægevej 16, DK-1870 Frederiksberg, Denmark; jw@sund.ku.dk (J.L.W.); anders.schroeder@sund.ku.dk (A.S.S.); caroline.gleerup@sund.ku.dk (C.H.G.); maiken.thode@sund.ku.dk (M.B.T.B.); 2 Anicura Dyreklinikken Telemark, Brennavn. 8, 3810 Gvarv, Norway; inge.engeland@anicura.no

**Keywords:** canine, degenerative mitral valve disease, dog, echocardiography, heart

## Abstract

**Simple Summary:**

Our study addresses a significant challenge in veterinary cardiology by improving the measurement of mitral regurgitation severity in dogs with myxomatous mitral valve disease, the most prevalent heart condition in dogs, particularly affecting breeds like Cavalier King Charles Spaniels and Chihuahuas. An accurate assessment of mitral regurgitation severity helps refine treatment decisions and disease monitoring, but the simple and reliable standard method still needs to be improved. This study evaluated 124 client-owned dogs using the proximal isovelocity surface area radius method (PISA-r). Our findings demonstrate that this measurement is accurate and consistent. We established specific threshold values for PISA-r important for determining the disease stage and making informed treatment choices. Notably, this measurement correlates strongly with other heart measurements, such as the left atrium size and left ventricular internal diameter, indicating its comprehensive assessment capability. The proximal isovelocity surface area radius increases significantly with disease progression. Statistical analysis confirms a high accuracy in classifying disease stages, with excellent sensitivity and specificity. Moreover, the PISA-r method exhibits excellent repeatability and reproducibility. Our research suggests that the PISA-r method is a valuable tool for diagnosing and managing dogs with myxomatous mitral valve disease.

**Abstract:**

Mitral regurgitation (MR) resulting from myxomatous mitral valve disease (MMVD) is a prevalent condition in dogs, particularly smaller breeds like Cavalier King Charles Spaniels (CKCSs) and Chihuahuas (CHHs). An accurate assessment of MR severity is essential for effective treatment and disease monitoring, yet a standardized method has yet to be established. In this retrospective study, we evaluated 124 client-owned dogs diagnosed with MMVD, including 64 CKCSs and 60 CHHs. Dogs were categorized into three stages: asymptomatic (B1), remodeled (B2), and congestive heart failure (CHF, C). The MR severity was quantified using the proximal isovelocity surface area (PISA) method, specifically focusing on the PISA radius (PISA-r). The PISA-r measurements exhibited significant increases across disease stages and demonstrated strong correlations with echocardiographic parameters (ranging from 0.83 to 0.94), including the left atrial size and left ventricular internal diameter. The receiver operating characteristic (ROC) curve analysis revealed the high sensitivity and specificity of PISA-r in classifying disease stages, establishing optimal cut-off values. The method displayed excellent repeatability (interobserver variability: 0.95) and reproducibility (intraobserver variability: 0.97). In conclusion, the PISA method, specifically PISA-r, was reliable for assessing MR severity in dogs with MMVD. By simplifying the diagnosis and management of MR, this research can potentially improve the life and management of MMVD-affected dogs.

## 1. Introduction

Myxomatous mitral valve disease (MMVD) is a leading cause of heart failure and cardiac death in middle-aged and older small to medium-sized dog breeds (<20 kg) [1]. The disease has a reported prevalence of 3.5% in dogs attending veterinary practices in England and is the third most common cause of death in dogs under 10 years of age [2,3]. Among all dog breeds, Cavalier King Charles Spaniels (CKCSs) are particularly susceptible to MMVD associated with mitral prolapse, with the disease often clinically evident at a younger age compared to other breeds. Chihuahuas (CHHs) are also predisposed to MMVD, but the disease typically appears later in life compared to its earlier onset in CKCSs [3,4,5,6,7,8,9,10].

Myxomatous changes to the mitral valve (MV) result in inadequate closure during systole, leading to a regurgitant blood flow jet from the left ventricle (LV) to the left atrium (LA). The progression of the valvular disease leads to eccentric hypertrophy, mitral annular dilation, and remodeling of the LA and LV as a compensatory response to the regurgitated volume [11]. Left-sided volume overload is the main clinical complication, leading to left-sided systolic congestive heart failure (CHF) associated with postcapillary pulmonary hypertension [1,12].

Administering pimobendan to dogs in the preclinical B2 stage of MMVD has been shown to extend the preclinical period and delay the onset of congestive heart failure or cardiac-related death [13]. Determining the optimal time to initiate treatment depends on the early detection of MMVD-associated MV lesions, assessing the severity of mitral regurgitation (MR), and evaluating its impact on cardiac remodeling [11]. Color Doppler echocardiography is commonly used to diagnose MR, while linear measurements from two-dimensional echocardiography are used to stage the disease by assessing LA and LV chamber sizes in dogs [1]. Volumetric methods are recommended in human medicine using the proximal isovelocity surface area (PISA) method [14,15]. This method has also shown superior results in evaluating MR severity in dogs compared to linear measurements [16]. While there are only a few studies on the use of the PISA method in dogs, it has been used to calculate the regurgitant fraction (RgF), effective regurgitation orifice area (EROA), and the ratio of MR to aortic flow rate (Qmr:Qao) in dogs [16,17,18,19,20,21,22].

The PISA method is recommended for evaluating MR severity in human cardiology, measuring EROA and the regurgitant volume (RgV) to assess lesion severity and volume overload, respectively [14,15,23]. This method utilizes a hydrodynamic principle where blood flow gradually increases as it approaches a circular hole [24]. The PISA refers to the aliasing hemisphere visible adjacent to the regurgitant orifice on the ventricular side, with a larger PISA indicating a larger regurgitant orifice. While the PISA method has shown potential in assessing dogs with MMVD [16,17,18,19,20,21,22], the current standard for evaluating MR severity in veterinary medicine is still based on measuring LA and LV sizes [11]. However, these measurements are not intended to assess the severity of MR, as severe MR can occur even in dogs with normal-sized LA and LV. While MMVD is the most common cause of MR in dogs, other conditions, such as endocarditis and dysplasia, can also lead to MR. Other methods, including those described by Laoruche-Lebel et al. (2019) and Muzzi et al. (2003), are used to evaluate MR severity [16,25,26,27]. The PISA method may be less commonly used due to its perceived complexity and lack of documentation in the veterinary literature [16].

A simplified PISA method based on measuring only the radius of the proximal isovelocity surface area (PISA-r) has been validated in human medicine and is recommended by the American Society of Echocardiography [28,29,30]. However, the potential contribution of PISA-r to assessing MR severity in dogs has yet to be investigated or validated.

This study aims to (1) evaluate the severity of MR in dogs with MMVD using echocardiographic derived PISA-r, (2) establish PISA-r cut-off values for the different stages of MMVD severity in accordance with the American College of Veterinary Internal Medicine (ACVIM) classification, and (3) investigate the relationship between PISA-r and key echocardiographic measurements, including the normalized left ventricular internal diameter in diastole (LVIDdN), the left atrium-to-aorta ratio (LA/AO), and the left atrium maximum volume (LAmax), in CKCSs and CHHs at various stages of MMVD [1].

## 2. Animals, Materials and Methods

This retrospective study was conducted at the University Hospital for Companion Animals, University of Copenhagen in 2020. The Ethical and Administrative Committee approved the study at the Department of Veterinary Clinical Sciences, University of Copenhagen, Denmark (EAU, 2019-19). Cavalier King Charles Spaniels and CHHs with and without MMVD were included from the Veterinary Teaching Hospital cardiac examination database and grouped according to the ACVIM staging system as described below.

### 2.1. Animals

Controls (Stage A) included CKCSs and CHHs with normal cardiac size and function and no detectable MR on color Doppler or a structural MV abnormality [1,31]. Dogs included as controls furthermore had to have an unremarkable medical history.

Stage B1 included dogs with MR that met either or both of the following criteria: LVIDdN < 1.7 or LA/Ao < 1.6.

Stage B2 included dogs with MR that met the criteria: LVIDdN ≥ 1.7 and LA/Ao ≥ 1.6.

Furthermore, no clinical signs or presence of CHF were present in dogs in stages B1 and B2 [1,31].

Stage C included dogs with MR that met the criteria of LVIDdN ≥ 1.7 and LA/Ao ≥ 1.6, and the findings were supported by clinical signs consistent with congestive heart failure, i.e., acute tachypnea, coughing, exercise intolerance, and abnormal thoracic auscultation with evidence of pulmonary edema on thoracic radiographs or lung ultrasounds showing more than 3 B-lines per view in several areas or a clinical response to diuretic treatment. A treatment protocol including furosemide before echocardiography was not an exclusion criterion.

Diagnostic criteria for MMVD included a left apical systolic murmur and MR detected on color Doppler in connection with thickened or prolapsed MV leaflets [32].

Inclusion in the study required a medical record and echocardiographic examination of sufficient quality (2D, M-mode, and Doppler studies), including the left apical four-chamber color Doppler cine loop of the MV. Furthermore, dogs with flail valve, multiple PISAs, evidence of other non-MMVD heart disease, or comorbidities that could affect the hemodynamic response (e.g., hypertension (>180 mmHg), primary respiratory disease, or kidney disease) could not be included in the study [15,33].

Cavalier King Charles Spaniels and CHHs meeting the inclusion criteria were retrospectively recruited from the database starting from 2020 and going backwards until a minimum of 15 CKCSs and 15 CHHs were recruited in each of the four groups. The clinically healthy dogs included in the control group (stage A) had an echocardiographic examination as a part of an established national screening program (CKCSs) or participated in a previous study group (all CHHs and some CKCSs) [34].

The heart rate for all dogs was obtained from the clinical medical record during the clinical examination after acclimatization. Auscultation and palpation over 30 s were performed to ensure consistency and avoid variations caused by echocardiographic procedures.

### 2.2. Echocardiography

All echocardiographic examinations were performed by experienced operators using a General Electric Vivid E9 ultrasonographic system with a 6S multi-frequency phased array probe. Raw data from all dogs were captured digitally for subsequent offline analysis using the EchoPac PC software (version 204, GE Medical Systems). Echocardiography was performed with simultaneous ECG, and all echocardiographic measurements were taken from three consecutive heart cycles and averaged.

Transthoracic echocardiographic views were performed based on the standardized imaging planes and display conventions recommended by Thomas et al. (1993). The right parasternal and left apical parasternal locations were utilized to obtain consistent two-dimensional images [35].

**Figure 1:**
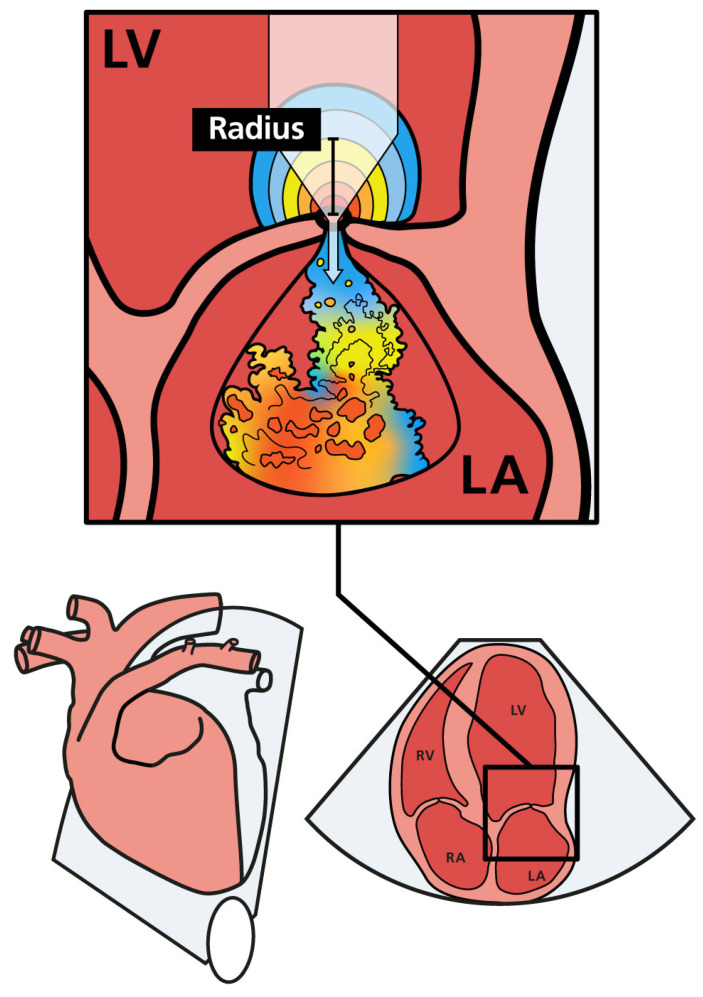
Schematic illustration of mitral regurgitation with PISA and the regurgitant jet in the left atrium. As regurgitant blood approaches the mitral orifice, the flow velocity increases. The flow forms a hemisphere with multiple distinct layers, each having an equal velocity (color coded). The PISA radius (PISA-r) is shown as a black bar. LA (left atrium); LV (left ventricle); RA (right atrium); RV (right ventricle).

PISA-r was measured using color flow Doppler from the apical four-chamber view. The view was initially focused on the MV region to obtain an optimal mitral regurgitant orifice. This view was magnified to identify the flow convergence region proximal to the regurgitant orifice. The baseline of the color flow scale was then shifted so that the Nyquist limit (or aliasing velocity) was decreased to a value of 35 cm/s in the flow direction by shifting the baseline of the color flow scale. This baseline shift was performed to visualize the hemispheric shell accurately, as described in the human literature [15]. Color flow was turned on and off to help identify the valve orifice. The PISA radius (r) was measured at mid-systole from the valve orifice to the point of color change (Figure 1) [24].

**Figure 2:**
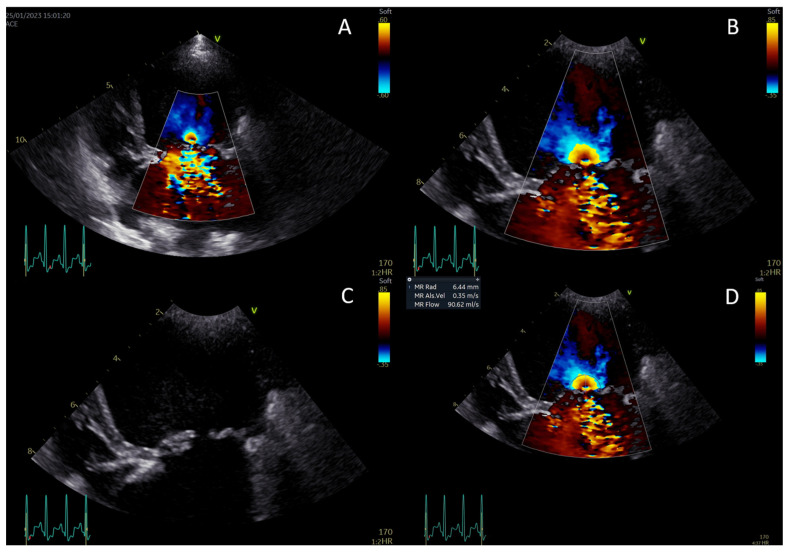
Measurement of the proximal isovelocity surface area (PISA) radius. Activate color Doppler and focus on the mitral valve (**A**). Zoom in and set the aliasing velocity to 35 cm/s, freeze the image, and scroll to mid-systole (**B**). Turn off the color Doppler to identify the valve orifice plane (**C**). Measure the PISA radius from the valve orifice to the dome of the hemisphere (**D**).

Continuous-wave Doppler was used to measure the maximal velocity of the regurgitant jet (V_max_) and the velocity–time integral. The PISA, EROA, and RgV were calculated, and RgF was then calculated by dividing the RgV by the stroke volume (Figure 2A–D) [13,21,22,29].

The dimensions of the LV cavity and wall thicknesses were measured using the leading edge-to-leading edge method on an M-mode view, guided at the level of the chordae tendineae in the right parasternal short-axis view [36]. The fractional shortening % (FS) and LVIDdN were automatically calculated from measurements of the LV internal dimensions in diastole (LVIDd) [37]. The left ventricle internal dimension in diastole was also measured in the 2D long-axis view to assess for potential off-axis bias. Inconsistency in measurements between the two views resulted in re-evaluating the patient’s LVIDd. If the off-axis bias was > 10%, the LVIDd from the 2D long-axis view was used.

The LA/Ao was measured according to the Swedish method [38], maximal and minimal LA volumes were measured using the biplane area–length method on the apical four-chamber view from the left side [34], and maximal LV and minimal LV volumes were measured using the monoplane Simpson’s method of discs (SMOD) performed on the apical four-chamber view from the left side [39]. The ejection fraction % (EF) and stroke volume (SV) were automatically calculated using the LV internal volume measurements.

The peak transmitral velocities (E and A) and aortic flow were assessed using pulsed-wave Doppler echocardiography from the apical four-chamber and five-chamber views. The peak velocity of the MR and tricuspid regurgitation (TR) were measured using continuous-wave Doppler (CWD) [40].

### 2.3. Statistical Analysis

All statistical analyses were performed using R version 4.3.0 (R core team 2023) [41]. The clinical and echocardiographic data collected from the dogs were presented as a medians with 2.5–97.5 percentiles. A *p*-value of <0.05 was considered statistically significant. The Kruskal–Wallis test was used to assess overall statistical significance between groups, whereas pairwise comparisons were performed using the Wilcoxon signed-rank test.

Correlations (r) (Pearson’s coefficient and linear regression) between PISA-r and the LA volume, LVIDdN, and LA/Ao were examined visually using scatter plots and statistically by linear regression. The correlation was considered perfect if the correlation coefficient was 1, strong between 0.7 and 0.9, moderate between 0.4 and 0.6, and weak < 0.3 [42].

Receiver operating characteristic (ROC) analyses were used to describe the ability of PISA-r to differentiate dogs in stage B2 from those in stage B1 and dogs in stage C from those in stage B2. ROC analyses were also performed to compare the additional clinical parameters, i.e., PISA-r, EROA, RgV, RgF, and LAmax (mL/kg). The overall ability of each parameter to correctly group the animals was assessed using the area under the curve (AUC). An AUC between 0.90 and 1 was excellent, while 0.80–0.90 was good, 0.70–0.80 was fair, and 0.60–0.70 was poor [43]. Sensitivity and specificity were calculated, and cut-off values were estimated for each parameter. The highest value when summarizing the specificity and sensitivity for each cut-off value was reported (Youden index).

Inter- and intraobserver variabilities for PISA-r were tested using the intraclass correlation coefficient (ICC). Two dogs from each MMVD group (a total of 12 dogs) were randomly selected, and PISA-r was measured by the original operator and a second operator to determine the interobserver variability. The original operator repeated the exact measurements a second time a week later to assess the intraobserver measurement variability [44,45]. A correlation coefficient greater than 0.8 indicates excellent agreement [46].

## 3. Results

**Figure 3:**
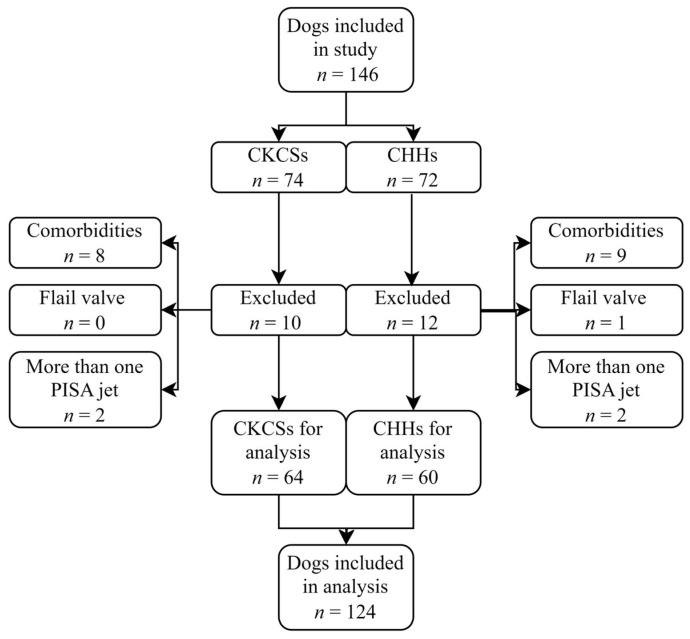
Flowchart of patient enrolment. An initial 146 Cavalier King Charles Spaniels (CKCSs) and Chihuahuas (CHHs) were included in the study. A total of 22 dogs were excluded, leaving 64 CKCSs and 60 CHHs for the final analysis.

A total of 146 client-owned dogs were evaluated in this study: 74 CKCSs and 72 CHHs. Of these, 22 dogs (15.1%) were excluded from the study based on the exclusion criteria, including 17 due to a comorbidity, 4 with more than one PISA jet, and 1 with a flail valve (Figure 3).

The inclusion criteria were therefore met by 124 (84.9%) of the 146 dogs that were included in this retrospective study. Of these, 64 were CKCSs, and 60 were CHHs. The study population included 92 dogs diagnosed with MMVD and 32 healthy control dogs. According to the ACVIM classification system, 17 CKCSs and 15 CHHs were classified as asymptomatic (stage B1), 15 CKCSs and 14 CHHs were classified as remodeled (stage B2), and 15 CKCSs and 16 CHHs were classified with CHF (stage C) (Table 1 and Table 2). Median estimates along with 2.5–97.5 percentiles of clinical characteristics are given in Table 1 and Table 2. The control dogs were significantly younger and weighed less than the dogs in the other groups. Most control dogs were female (24 compared to 8), and most dogs with MMVD were male (61 compared to 31). Heart rate was significantly increased in dogs with stage C MMVD compared to the control dogs.

Five of the 17 CKCSs (29.4%) and five of the 15 CHHs (33.3%) in stage B1, as well as six of the 15 CKCSs (40.0%), and four of the 14 CHHs (28.6%) in stage B2, were being treated with pimobendan at presentation. All the CKCSs (100%) and ten out of 15 CHHs (66.6%) in stage C were treated with pimobendan alone or in combination with an angiotensin-converting enzyme (ACE) inhibitor and spironolactone at presentation (Table 1 and Table 2).

The echocardiographic measurements are presented as medians with 2.5–97.5 percentiles in Table 1 and Table 2. The PISA-r in CKCSs ranged from 1.50 mm to 11.20 mm and from 1.66 mm to 8.12 mm in CHHs. The PISA-r increased significantly between stage B1 and B2 and between stage B2 and C for both breeds.

The PISA variables (EROA, RgV, and RgF) in stage B2 were significantly increased compared to stage B1 for both breeds. There were also significant increases in RgV and RgF for both CKCSs and CHHs in stage C compared to stage B2, as well as an increase in EROA between stage B2 and stage C. However, this increase was not significant (Table 1 and Table 2).

**Figure 4:**
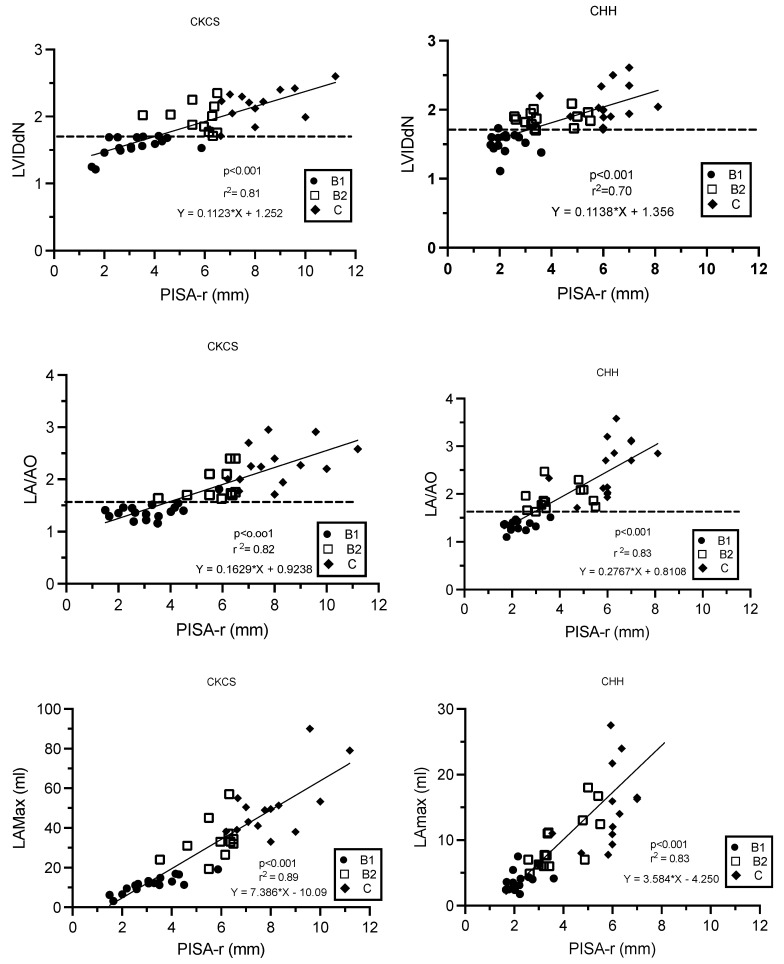
Linear regression models describing the relationships between PISA-r and LVIDdN, LA/AO ratio, and LAmax (mL), respectively, in Cavalier King Charles Spaniels and Chihuahuas with myxomatous mitral valve disease stage B1, B2, and C. The dotted line defines the cut-off values for LVIDdN (1.7) and LA/AO (1.6). B1 (stage B1); B2 (stage B2); C (stage C); CHH (Chihuahua); CKCS (Cavalier King Charles Spaniel); LA/AO (ratio of the left atrium/aorta); LAmax (left atrium maximum volume); LVIDdN (normalized left ventricular internal diameter in diastole); MR (mitral regurgitation); PISA-r (radius of the proximal isovelocity surface area); r^2^ (coefficient of determination).

There were linear relationships between PISA-r and LVIDdN, LA/Ao, and LAmax (mL), as described by the regression formulas (Figure 4). As LA/Ao, LVIDdN, and LAmax increased, PISA-r increased linearly (*p* < 0.001, Figure 4). The correlation coefficients were strong for all variables and varied from 0.83 to 0.94.

**Figure 5:**
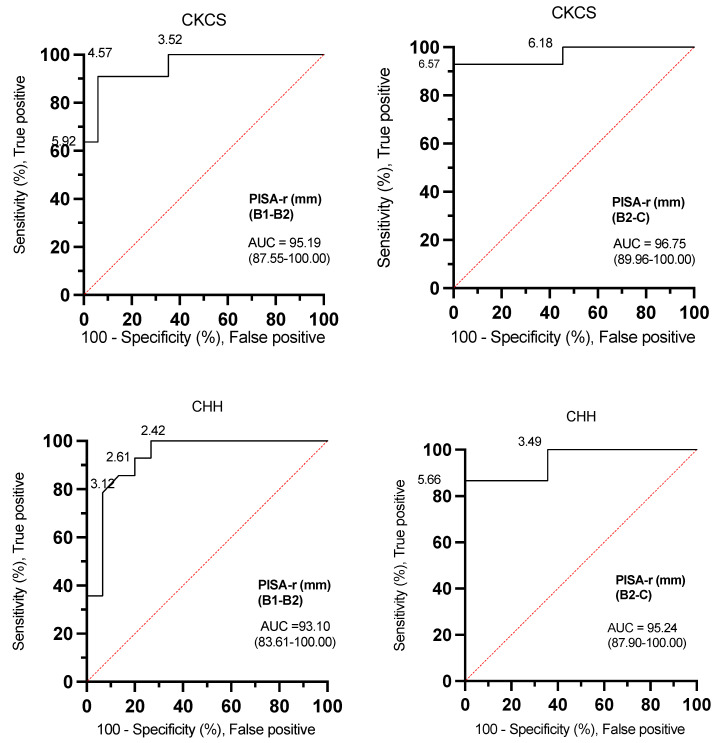
Receiver operating characteristic (ROC) curves with PISA-r cut-off values to differentiate dogs with stage B1 or stage B2 mitral valve disease and dogs with stage B2 or stage C mitral valve disease based on the desired sensitivity and specificity. AUC (area under the curve); B1 (stage B1); B2 (stage B2); C (stage C); CKCS (Cavalier King Charles Spaniel); CHH (Chihuahua); PISA-r (radius of the proximal isovelocity surface area).

The ROC curve analyses for PISA-r were excellent for both CKCSs and CHHs (AUC: 93.10–96.75) when determining whether a dog was classified B1, B2, or C (Figure 5, Table 3).

The ROC curves effectively demonstrate the versatility of determining optimal threshold settings. In the context of canine MMVD, the ROC analysis revealed that a PISA-r < 6.57 mm, exhibiting a sensitivity of 92.86% and specificity of 100% in CKCSs, along with a PISA-r < 5.66 mm, featuring a sensitivity of 86.67% and specificity of 100% in CHHs represent the cut-off values with maximum sensitivity and specificity for excluding congestive heart failure (CHF, stage C). Similarly, to exclude remodeling (stage B2) in MMVD-afflicted dogs, the ROC analysis identified a PISA-r < 4.57 mm with a sensitivity of 91% and specificity of 94% in CKCSs, and a PISA-r < 2.42 mm with a sensitivity of 100% and specificity of 73% in CHHs (refer to Table 3 for a summary of cut-off values for indexed LAmax (mL/kg), EROA, RgV, and RgF).

The ICC showed excellent repeatability and reproducibility for measuring PISA-r, as the interobserver variability was 0.95 (0.84–0.99) and intraobserver variability was 0.97 (0.88–0.99).

## 4. Discussion

This retrospective study investigated using PISA-r to assess the severity of MR in CKCSs and CHHs with MMVD. Previous studies have reported using the PISA method to assess MR severity in dogs [18,19,22]. Our study revealed that PISA-r increases with worsening disease severity, and linear relationships between PISA-r and LVIDdN, LA/Ao, and LAmax (mL) were found. Cut-off values distinguishing stages B1 and B2, as well as B2 and C, were established for PISA-r, EROA, RgV, RgF, and indexed LAmax (mL/kg) in CHHs and CKCSs with MMVD.

### 4.1. PISA and PISA-r

For both CKCSs and CHHs, PISA-r could differentiate MMVD stages (B1 vs. B2 and B2 vs. C), and cut-off values with a high sensitivity and specificity were established for both breeds (Table 3).

To further quantify MR severity in dogs with MMVD, the maximum and minimal volumes of LV, velocity time integral of the MR, and maximal MR flow were measured and used in the calculations of EROA, RgV, and RgF. Our findings suggest an increase in all these variables with increasing MR severity. This is in accordance with similar findings from previous studies [17,21,29].

PISA-r enables a rapid estimation of MR severity. PISA-r is measured and used in the calculation of PISA, EROA, and RgV. However, PISA-r is often not reported or discussed in studies evaluating MR severity. Assessing the severity through PISA-r makes it less pivotal to measure and calculate the other parameters necessary to obtain EROA and RgV. PISA-r is less time-consuming and avoids measurement errors that otherwise would be squared due to the formulas used in the calculations.

The PISA method, despite being widely used in human cardiology and less in veterinary medicine, has several limitations in the assessment of MR severity [16,19,47]. Firstly, the PISA calculation assumes a hemispheric flow convergence zone, circular EROA, centrally directed flow, a static mitral orifice shape, and a holosystolic regurgitation [14], which often lead to an underestimation of the MR severity in cases where the orifice shape is elliptical and not circular [48]. Moreover, the assumption of a static orifice shape is not valid in cases of MV prolapse, where the shape is complex and dynamic, leading to difficulties in determining the severity of MR [49]. Secondly, the limitations of PISA are compounded when the hemisphere is incomplete due to flow restriction by the mitral leaflets or the ventricular wall, leading to an overestimation of the MR severity. Additionally, the method cannot be used in cases of multiple regurgitant orifices. In veterinary medicine, the use of PISA to assess MR in dogs with MMVD has been found to be repeatable, reproducible, useful, and correlated with disease severity [18,20,21,50]. However, the method is challenging to utilize routinely due to difficulties in measurement and the requirement for high image quality and offline analysis [16]. The PISA-r method can be a valuable adjunct to traditional echocardiographic parameters for assessing MR severity in clinical practice. Its robust correlations with key volumetric parameters such as LAmax, LA/AO, and LVIDdN enhance diagnostic accuracy and inform treatment decisions. For instance, in cases where dogs present with comorbidities such as pulmonary disease, e.g., airway obstruction, making it difficult to distinguish the cause of dyspnea, PISA-r can provide an additional insight into the underlying MR severity. Adopting PISA-r may represent a significant advancement in MR evaluation, offering clinicians a valuable adjunctive tool to refine their diagnostic approach and monitor and optimize patient care.

In general, dogs with MMVD are clinically classified according to the ACVIM guidelines, where LA/AO and LVIDdN are used to define cardiac remodeling. However, in this classification scheme, dogs with mild, moderate, and severe MR may all be classified as stage B2.

### 4.2. Clinical Evaluation

The clinical evaluation revealed a predominance of male dogs with MMVD, consistent with findings from prior studies. Additionally, dogs with MMVD were significantly older compared to control dogs (Table 1 and Table 2), aligning with the well-established association between MMVD prevalence and advancing age [50]. Notably, CKCSs exhibited an earlier onset of MMVD, typically at three years of age, compared to CHHs at six years, consistent with the existing literature [2,4,5,6].

Despite current recommendations advising against treatment for dogs classified as stage B1, our study identified five CKCSs and five CHHs categorized as B1 who were undergoing treatment with pimobendan upon presentation [1]. A subsequent review of their medical records revealed that all these dogs had been previously diagnosed as stage B2 during an earlier echocardiographic assessment, prompting the initiation of treatment in accordance with the guidelines. Notably, it has been demonstrated that pimobendan treatment effectively reduces cardiac dimensions, suggesting that dogs initially diagnosed as stage B2 may exhibit regression to stage B1 upon a subsequent echocardiographic evaluation [13].

### 4.3. MR Severity

ROC curves were constructed to identify echocardiographic variables and help predict a greater risk of remodeling (stage B2 vs. B1). PISA-r, LAmax (mL/kg), EROA, RgV, and RgF were selected for the ROC analyses. All AUC values > 91.98% indicated a high discriminatory ability (Figure 5 and Table 3). The current understanding regarding the utility of PISA-r, EROA, RgV, and RgF cut-off values in identifying dogs at risk of future left-sided heart enlargement remains less clear.

The risk of cardiac remodeling (i.e., the risk of being classified in stage B2) increases as the PISA-r increases toward 5.8 mm, EROA toward 0.15 cm^2^, RgV toward 13.88 mL and RgF toward 51.6% in the CKCSs. The same values for the CHHs are a PISA-r of 3.84 mm, EROA of 0.068 cm^2^, RgV of 5.49 mL, and RgF of 57.3%. Therefore, measuring PISA-r, EROA, RgV, or RgF in dogs with preclinical MMVD can aid in assessing MR severity, especially in B2 dogs.

A diagnosis of CHF is normally based on a combination of clinical signs, including exercise intolerance, respiratory distress, tachypnea, coughing and moist respiratory sounds, and radiographs showing pulmonary edema or B-lines on a lung ultrasound [51,52]. The AUCs of PISA-r were 96.75% and 95.24%, differentiating stage C from stage B2 for CKCSs and CHHs, respectively. A PISA-r of less than 6.50 mm (sensitivity of 92.86% and specificity of 100%) in CKCSs and less than 5.50 mm (sensitivity of 86.67% and specificity of 100%) in CHHs can, based on this study, be used as cut-off values for excluding CHF in individual dogs. However, these cut-off values may not be pathophysiologically correct due to variations in the individual dog’s capacity to compensate for MR and the speed at which the disease progresses.

Compared to CHHs, the higher median mitral inflow E-wave velocity in CKCSs with stage B2 MMVD reflects more advanced cardiac remodeling in CKCSs (Table 1 and Table 2). The CKCSs have larger LA volumes (3.37 mL/kg vs. 2.68 mL/kg) and higher LVIDdN (2.01 vs. 1.86), indicating higher LA pressure and volume overload due to more severe MR. This results in elevated transmitral pressure gradients and higher E-wave velocities. With lower velocities and lower minimum range values, CHHs may be in an earlier phase of stage B2, with less severe atrial and ventricular changes. Thus, CKCSs appears to be in a later stage of B2, possibly reflecting breed-specific disease progression

### 4.4. Clinical Applications

Increased LA/Ao ratios and LVIDdN values in dogs with MMVD suggest chronic volume overload of the LA and LV, as MR leads to an elevated pressure and volume in these chambers. These parameters, however, only indirectly reflect the chronic effects of MR on the heart’s left side [53,54].

In comparison, PISA-r provides a direct measurement of MR severity by quantifying the regurgitant flow, offering a more accurate assessment of the dynamic nature of MR progression [53]. This method captures the regurgitant volume in real-time, making it sensitive to acute changes in loading conditions or complications. It can be repeated over time to follow disease progression more precisely. As a result, it enables earlier and more accurate assessments of MR progression [15,54].

Additionally, in cases of pulmonary edema, especially in late MMVD stage B2 dogs with concurrent conditions like tracheal collapse, PISA-r may help differentiate whether the edema is primarily due to respiratory issues or heart failure, aiding in targeted treatment strategies.

### 4.5. Limitations of the Study

Our study is limited by the focus on only two breeds, potentially constraining the generalizability of our findings. Future investigations should encompass larger and more diverse populations, including multiple breeds, to ascertain the broader applicability of our results. A potential limitation of our study is the imbalance in sex distribution, with more females in the control group and more males in the affected group. This may have influenced some echocardiographic measurements, as males are often larger than females, which could affect specific echocardiographic parameters.

The exclusion of dogs and subsequent regrouping after data collection resulted in varying group sizes, potentially influencing the study outcomes. Additionally, our study lacked a comparison to a gold standard method for MR severity assessment.

Furthermore, medication effects, particularly from commonly used ACE inhibitors and spironolactone in heart failure management, could have influenced echocardiographic indices. The decision to withhold medications might have altered the observed results.

## 5. Conclusions

In conclusion, our study demonstrates the utility of PISA-r as a volume-based parameter in assessing MMVD severity in dogs. Its ease of measurement and reproducibility make it a valuable tool, supported by established cut-off values for group affiliation. Serial PISA-r assessments offer insights into disease progression and treatment efficacy, complementing other parameters like LAmax, LA/Ao, and LVIDdN for enhanced accuracy in the MMVD stage classification.

## Figures and Tables

**Table 1:** Demographic and echocardiographic variables of 64 Cavalier King Charles Spaniel dogs. A total of 17 control dogs and 47 dogs affected with myxomatous mitral valve disease at different stages (B1, B2, and C) were included.

	Control CKCSs (*N* = 17)		CKCSs with MMVD B1 (*N* = 17)		CKCSs with MMVD B2 (*N* = 15)		CKCSs with MMVD C (*N* = 15)		*p*
On treatment at presentation (nb)	0 (0.0%)		5 (29.4%)		6 (40.0%)		14 (93.3%)		-
Age (years)	2.0	(1.0–4.4) ^a^	6.0	(3.2–11.5) ^b^	8.5	(6.6–10.1) ^b^	9.0	(6.4–12.1) ^b^	<0.001
Male/Female (nb)	3 (17.6%)/14 (82.4%)		11 (64.7%)/6 (35.3%)		9 (60.0%)/6 (40.0%)		11 (73.3%)/4 (26.7%)		-
Body weight (Kg)	7.8	(5.0–10.5) ^a^	11.3	(7.8–14.6) ^b^	10.10	(7.3–11.9) ^b^	9.2	(7.5–14.6) ^b^	<0.001
Heart rate	125	(100–148) ^a^	140	(107–160) ^ab^	140	(96–165) ^ab^	156	(105–182) ^b^	<0.021
IVSd (mm)	6.36	(5.11–9.09) ^a^	7.86	(6.45–10.73) ^b^	7.33	(5.85–9.94) ^ab^	6.68	(5.46–11.20) ^ab^	<0.001
LVIDd (mm)	24.94	(20.40–29.13) ^a^	32.51	(22.50–36.20) ^b^	38.18	(31.20–45.34) ^bc^	42.82	(33.00–50.25) ^c^	<0.001
LVPWd (mm)	6.52	(4.76–7.00) ^a^	7.17	(4.99–9.33) ^b^	7.27	(5.70–8.57) ^b^	7.34	(5.11–9.10) ^b^	0.002
IVSs (mm)	8.33	(6.19–11.02) ^a^	10.33	(7.99–12.49) ^b^	10.14	(7.90–14.16) ^b^	11.32	(8.66–14.70) ^b^	<0.001
LVIDs (mm)	16.24	(10.80–20.90) ^a^	20.14	(11.93–22.36) ^ab^	23.00	(16.31–31.00) ^b^	24.33	(15.72–29.30) ^b^	<0.001
LVPWs (mm)	9.67	(6.88–11.82) ^a^	10.97	(8.61–14.52) ^b^	12.00	(8.70–15.10) ^b^	11.95	(8.92–14.70) ^b^	<0.001
LV FS (%)	34.3	(21.7–50.9) ^a^	37.2	(32.2–51.3) ^a^	40.0	(32.0–51.2) ^ab^	44.7	(32.7–54.4) ^b^	0.012
LVIDdN	1.34	(1.13–1.58) ^a^	1.56	(1.23–1.71) ^a^*	2.01	(1.71–2.31) ^b^*	2.22	(1.74–2.54) ^b^	<0.001
LVEDV (mL)	12.00	(7.87–16.53) ^a^	21.33	(12.47–25.20) ^a^	31.00	(24.00–56.10) ^b^	39.00	(25.25–56.55) ^b^	<0.001
LVEDV (mL/kg)	1.57	(1.02–2.04) ^a^	1.75	(1.31–2.41) ^a^	3.70	(2.60–5.10) ^b^	3.89	(2.49–6.26) ^b^	<0.001
LVESV (mL)	3.33	(2.00–6.73) ^a^	6.00	(2.53–7.33) ^a^	9.00	(4.01–19.00) ^b^	9.33	(3.95–15.10) ^b^	<0.001
LVESV (mL/kg)	0.46	(0.21–0.80) ^a^	0.53	(0.29–0.65) ^a^	1.03	(0.48–2.10) ^b^	0.93	(0.46–1.63) ^b^	<0.001
LV EF (%)	71.1	(57.1–79.1) ^a^	70.2	(60.1–82.8) ^ab^	72.0	(49.1–86.0) ^ab^	76.1	(66.6–84.7) ^b^	0.170
LA max (mL)	5.32	(2.63–6.65) ^a^	11.77	(4.32–18.26) ^ab^	32.00	(17.12–53.00) ^bc^	49.00	(30.72–86.15) ^c^	<0.001
LA max (mL/kg)	0.62	(0.36–0.97) ^a^	1.02	(0.46–1.54) ^a^	3.37	(2.16–5.04) ^b^	4.75	(2.99–8.23) ^c^	<0.001
LA min (mL)	1.56	(0.88–2.60) ^a^	3.79	(1.52–6.05) ^a^	13.80	(5.12–24.78) ^b^	28.31	(15.60–52.90) ^c^	<0.001
LA min (mL/kg)	0.22	(0.13–0.33) ^a^	0.34	(0.15–0.53) ^a^	1.57	(0.60–2.23) ^b^	3.02	(1.32–4.96) ^c^	<0.001
LA/Ao	1.23	(1.00–1.36) ^a^	1.38	(1.18–1.70) ^a^*	1.73	(1.62–2.40) ^b^*	2.25	(1.73–2.94) ^b^	<0.001
PISA-r (mm)	-	-	3.07	(1.56–5.32) ^a^	5.84	(4.00–6.50) ^b^	8.00	(6.35–10.78) ^c^	<0.001
MRVTI (cm)	-	-	88.90	(50.58–114.99) ^a^	98.4	(80.60–127.00) ^b^	74.43	(57.77–95.77) ^a^	0.002
Peak MR (m/s)	-	-	5.88	(5.26–7.06) ^a^	5.80	(5.28–6.38) ^a^	5.34	(1.60–5.97) ^b^	0.012
EROA (cm^2^)	-	-	0.034	(0.009–0.11) ^a^	0.15	(0.08–0.23) ^b^	0.27	(0.17–0.46) ^b^	<0.001
RgV (mL)	-	-	3.18	(0.51–9.44) ^a^	11.8	(6.35–20.00) ^b^	21.00	(12.33–34.65) ^c^	<0.001
RgF (%)	-	-	21.6	(5.8–54.8) ^a^	50.0	(33.0–83.0) ^b^	70.0	(54.4–94.0) ^c^	<0.001
Peak E (m/s)	0.67	(0.51–0.86)^a^	0.80	(0.58–0.95) ^a^	1.47	(1.16–1.71) ^b^	1.75	(1.17–2.21) ^c^	<0.001
Peak TR (m/s) *	-	-	2.31	(2.0–2.80) ^a^ n = 12	2.87	(2.06–3.11) ^ab^ n = 12	3.26	(2.81–4.06) ^b^ n = 14	<0.001

Numerical data are presented as medians with 2.5–97.5 percentiles. Different letters in the same row represent a statistical difference. Note: LVIDdN and LA/Ao were used to subclassify dogs with MMVD stage B to stages B1 and B2. The statistically significant difference between these measurements was caused by bias. * Reduced number of dogs. Ao (aorta); EF (ejection fraction); EROA (effective regurgitation orifice area); FS (fractional shortening); IVSd (interventricular septal thickness at end-diastole); IVSs (interventricular septal thickness at end-systole); LA (left atrium); LAmax (maximal volume of the left atrium); LV (left ventricle); LVEDV (left ventricular end-diastolic volume); LVESV (left ventricular end-systolic volume); LVIDd (left ventricular internal diameter in diastole); LVIDs (left ventricular internal diameter in systole); LVIDdN (normalized left ventricular internal diameter in diastole); LVPWd (left ventricular posterior wall at end-diastole); LVPWs (left ventricular posterior wall at end-systole); MMVD (myxomatous mitral valve disease); MR (mitral regurgitation); MRVTI (mitral regurgitation velocity time integral); MV (mitral valve); PISA (proximal isovelocity surface area); RgF (regurgitant fraction); RgV (regurgitant volume); TR (tricuspid regurgitation).

**Table 2:** Demographic and echocardiographic characteristics of 60 Chihuahua dogs. A total of 15 control dogs and 45 dogs affected with myxomatous mitral valve disease at different stages (B1, B2, and C) were included.

	Control CHHs (*N* = 15)		CHHs with MMVD B1 (*N* = 15)		CHHs with MMVD B2 (*N* = 14)		CHHs with MMVD C (*N* = 16)		*p*
On treatment at presentation (nb)	0 (0.0%)		5 (33.3%)		4 (28.6%)		10 (62.5%)		-
Age (years)	2.5	(0.9–8.0) ^a^	9.5	(6.0–11.3) ^b^	11.0	(8.7–16.4) ^c^	10.0	(6.4–14.5) ^bc^	<0.001
Male/Female (nb)	5 (33.3%)/10 (66.6%)		9 (60.0%)/6 (40.0%)		9 (64.3%)/5 (35.7%)		12 (75.0%)/4 (25.0%)		-
Body weight (kg)	2.3	(1.7–3.3) ^a^	3.2	(2.0–4.6) ^ab^	3.2	(2.1–5.0) ^b^	4.3	(2.1–5.6) ^b^	<0.001
Heart rate	118	(84–146) ^a^	140	(107–158) ^a^	136	(103–167) ^a^	155	(112–204) ^b^	<0.001
IVSd (mm)	4.51	(3.66–5.61) ^a^	5.03	(3.21–6.77) ^a^	4.95	(3.64–6.20) ^a^	5.00	(3.56–6.75) ^a^	0.444
LVIDd (mm)	18.02	(14.66–20.83) ^a^	21.00	(17.43–24.65) ^b^	26.00	(23.14–30.43) ^c^	30.00	(23.46–39.39) ^d^	<0.001
LVPWd (mm)	4.73	(3.33–5.75) ^a^	4.40	(3.16–5.98) ^a^	5.35	(3.48–6.61) ^a^	5.64	(3.54–6.58) ^a^	0.037
IVSs (mm)	6.32	(5.14–9.60) ^a^	7.36	(6.06–10.30) ^ab^	8.81	(6.95–10.40) ^b^	9.94	(6.33–11.02) ^b^	<0.001
LVIDs (mm)	11.04	(8.15–12.45) ^a^	11.33	(8.23–15.67) ^ac^	13.57	(10.29–18.18) ^bc^	14.68	(10.12–22.14) ^b^	<0.001
LVPWs (mm)	7.55	(6.34–8.82) ^a^	8.00	(6.41–10.00) ^ab^	9.00	(7.61–11.32) ^b^	9.12	(6.43–10.78) ^b^	<0.002
LV FS (%)	41.5	(27.0–52.0) ^a^	47.2	(33.9–54.9) ^a^	48.6	(36.0–59.1) ^a^	47.8	(34.9–62.9) ^a^	0.084
LVIDdN	1.38	(1.20–1.58) ^a^	1.59	(1.20–1.70) ^a^*	1.86	(1.70–2.07) ^b^*	2.00	(1.72–2.57) ^b^	<0.001
LVEDV (mL)	4.00	(3.00–7.00) ^a^	7.33	(4.00–10.20) ^ab^	11.00	(6.55–18.90) ^bc^	13.17	(8.12–30.00) ^c^	<0.001
LVEDV (mL/kg)	1.62	(1.11–3.23) ^a^	2.22	(1.28–2.79) ^a^	3.61	(2.17–4.91) ^b^	4.22	(2.22–6.46) ^b^	<0.001
LVESV (mL)	1.00	(1.00–2.00) ^a^	1.67	(1.00–3.43) ^ab^	2.00	(1.00–3.23) ^b^	3.00	(1.00–7.25) ^b^	0.001
LVESV (mL/kg)	0.53	(0.31–1.02) ^a^	0.54	(0.31–0.99) ^a^	0.63	(0.36–1.12) ^a^	0.73	(0.36–1.69) ^a^	0.208
LV EF (%)	70.0	(60.9–81.7) ^a^	75.0	(63.6–85.5) ^ab^	82.9	(64.7–88.8) ^b^	82.7	(71.0–88.6) ^b^	<0.001
LA max (mL)	1.75	(0.76–2.34) ^a^	3.61	(1.98–7.12) ^a^	7.69	(5.25–17.59) ^b^	15.00	(7.85–34.25) ^c^	<0.001
LA max (mL/kg)	0.72	(0.44–1.08) ^a^	1.16	(0.71–1.85) ^a^	2.68	(1.35–4.34) ^b^	4.26	(1.68–7.80) ^c^	<0.001
LA min (mL)	0.47	(0.20–0.86) ^a^	0.82	(0.55–2.27) ^a^	2.40	(1.42–7.50) ^b^	5.62	(2.18–18.17) ^b^	<0.001
LA min (mL/kg)	0.19	(0.12–0.42) ^a^	0.26	(0.16–0.54) ^a^	0.85	(0.32–2.24) ^b^	1.68	(0.46–3.91) ^b^	<0..001
LA/Ao	1.23	(0.96–1.36) ^a^	1.36	(1.15–1.50) ^a^*	1.84	(1.64–2.41) ^b^*	2.52	(1.73–3.44) ^c^	<0.001
PISA-r (mm)	-	-	2.15	(1.67–3.40) ^a^	3.38	(2.59–5.48) ^b^	6.00	(4.00–7.73) ^c^	<0.001
MRVTI (cm)	-	-	81.90	(43.25–101.99) ^a^	89.28	(67.86–124.41) ^a^	74.58	(55.50–92.63) ^b^	0.016
Peak MR (m/s)	-	-	6.14	(4.75–6.64) ^a^	576.50	(450.38–640.13) ^a^	567.50	(439.13–648.13) ^a^	1.115
EROA (cm^2^)	-	-	0.017	(0.010–0.039) ^a^	0.051	(0.025–0.12) ^b^	0.17	(0.02–0.20) ^b^	<0.001
RgV (mL)	-	-	1.26	(0.65–3.42) ^a^	4.43	(2.18–10.06) ^b^	10.96	(4.02–17.63) ^c^	<0.001
RgF (%)	-	-	30.1	(14.4–46.3) ^a^	56.0	(29.3–84.6) ^b^	80.0	(46.5–100.0) ^c^	<0.001
Peak E (m/s)	0.64	(0.50–0.76) ^a^	0.86	(0.63–1.02) ^a^	1.29	(0.74–1.76) ^b^	1.72	(1.19–2.09) ^c^	<0.001
Peak TR (m/s) *	-	-	2.76	(2.23–3.93) ^a^ n = 4	2.74	(2.2–3.68) ^a^ n = 9	3.31	(2.0–4.61) ^a^ n = 13	<0.001

Numerical data are presented as medians with 2.5–97.5 percentiles. Different letters in the same row represent a statistical difference. Note: LVIDdN and LA/Ao were used to subclassify dogs with MMVD stage B in stages B1 and B2. The statistically significant difference between these measurements was caused by bias. * Reduced number of dogs. Ao (aorta); EF (ejection fraction); EROA (effective regurgitation orifice area); FS (fractional shortening); IVSd (interventricular septal thickness at end-diastole); IVSs (interventricular septal thickness at end-systole); LA (left atrium); LAmax (maximal volume of the left atrium); LV (left ventricle); LVEDV (left ventricular end-diastolic volume); LVESV (left ventricular end-systolic volume); LVIDd (left ventricular internal diameter in diastole); LVIDs (left ventricular internal diameter in systole); LVIDdN (normalized left ventricular internal diameter in diastole); LVPWd (left ventricular posterior wall at end-diastole); LVPWs (left ventricular posterior wall at end-systole); MMVD (myxomatous mitral valve disease); MR (mitral regurgitation); MV (mitral valve); MRVTI (mitral regurgitation velocity time integral); PISA (proximal isovelocity surface area); RgF (regurgitant fraction); RgV (regurgitant volume); TR (tricuspid regurgitation).

**Table 3:** Area under the curves and corresponding cut-off values of selected echocardiographic variables in CKCS and CHH dogs to determine whether a dog was classified into MMVD stage B1 (A), stage B2 (B), or stage C (C).

	AUC (%)	95% CI	Cut-off Value	Se (%)	Sp (%)	*p*
Control vs. MMVD stage B1						
CKCS						
LAmax (mL/kg)	86.33	(72.53–100.00)	0.80	82.35	88.24	<0.001
CHH						
LAmax (mL/kg)	91.78	(80.70–100.00)	0.83	93.33	86.67	<0.001
MMVD stage B1 vs. stage B2						
CKCS						
PISA-r (mm)	95.19	(87.55–100.00)	4.57	90.91	94.12	<0.001
EROA (cm^2^)	95.99	(89.39–100.00)	0.090	90.91	94.12	<0.001
RgV (mL)	97.33	(92.25–100.00)	5.88	100.00	88.24	<0.001
RgF (%)	92.0	(81.3–100.0)	32.4	100.00	82.35	<0.001
LAmax (mL/kg)	100.00	(100.00–100.00)	1.97	100.00	100.00	<0.001
CHH						
PISA-r (mm)	93.10	(83.61–100.00)	2.42	100.00	73.33	<0.001
EROA (cm^2^)	96.19	(90.30–100.00)	0.024	100.00	80.00	<0.001
RgV (mL)	96.67	(91.08–100.00)	2.73	92.86	93.33	<0.001
RgF (%)	92.3	(82.1–100.0)	38.0	92.86	80.00	<0.001
LAmax (mL/kg)	96.43	(90.54–100.00)	1.53	92.86	86.67	<0.001
MMVD stage B2 vs. stage C						
CKCS						
PISA-r (mm)	96.75	(89.96–100.00)	6.57	92.86	100.00	<0.001
EROA (cm^2^)	87.66	(74.19–100.00)	0.165	100.00	63.64	0.002
RgV (mL)	82.79	(66.69–98.89)	20.50	64.29	100.00	0.005
RgF (%)	79.6	(60.0–99.1)	52.0	100.00	63.64	0.013
LAmax (mL/kg)	87.34	(72.94–100.00)	4.27	78.57	90.91	0.002
CHH						
PISA-r (mm)	95.24	(87.90–100.00)	5.66	86.67	100.00	<0.001
EROA (cm^2^)	82.62	(66.86–98.38)	0.134	60.00	100.00	0.003
RgV (mL)	88.10	(75.73–100.00)	9.55	73.33	92.86	<0.001
RgF (%)	81.0	(65.1–96.9)	67.5	80.00	78.57	0.005
LAmax (mL/kg)	75.71	(57.53–93.90)	4.21	60.00	92.86	0.018

*p*-values for the corresponding univariate regression models are shown. AUC (area under the curve); CKCS (Cavalier King Charles Spaniel); CHH (Chihuahua); EROA (effective regurgitant orifice area); LA (left atrium); LAmax (maximal volume of left atrium); MMVD (myxomatous mitral valve disease); PISA-r (radius of the proximal isovelocity surface area); RgF (regurgitant fraction); RgV (regurgitant volume); Se (sensitivity); Sp (specificity).

## Data Availability

Data are unavailable due to ongoing research activities.

## References

[1] Keene B.W., Atkins C.E., Bonagura J.D., Fox P.R., Haggstrom J., Fuentes V.L., Oyama M.A., Rush J.E., Stepien R., Uechi M. (2019). ACVIM consensus guidelines for the diagnosis and treatment of myxomatous mitral valve disease in dogs. J. Vet. Intern. Med..

[2] Bonnett B.N., Egenvall A., Olson P., Hedhammar A. (1997). Mortality in insured Swedish dogs: Rates and causes of death in various breeds. Vet. Rec..

[3] Mattin M.J., Boswood A., Church D.B., Lopez-Alvarez J., McGreevy P.D., O’Neill D.G., Thomson P.C., Brodbelt D.C. (2015). Prevalence of and risk factors for degenerative mitral valve disease in dogs attending primary-care veterinary practices in England. J. Vet. Intern. Med..

[4] Beardow A.W., Buchanan J.W. (1993). Chronic mitral valve disease in cavalier King Charles spaniels: 95 cases (1987–1991). J. Am. Vet. Med. Assoc..

[5] Chetboul V., Tissier R., Villaret F., Nicolle A., Dean E., Benalloul T., Pouchelon J.L. (2004). Epidemiological, clinical, echo-doppler characteristics of mitral valve endocardiosis in Cavalier King Charles in France: A retrospective study of 451 cases (1995 to 2003). Can. Vet. J..

[6] Haggstrom J., Hansson K., Kvart C., Swenson L. (1992). Chronic valvular disease in the cavalier King Charles spaniel in Sweden. Vet. Rec..

[7] Pedersen H.D., Lorentzen K.A., Kristensen B.O. (1999). Echocardiographic mitral valve prolapse in cavalier King Charles spaniels: Epidemiology and prognostic significance for regurgitation. Vet. Rec..

[8] Thrusfield M.V., Aitken C.G.G., Darke P.G.G. (1985). Observations on Breed and Sex in Relation to Canine Heart-Valve Incompetence. J. Small Anim. Pract..

[9] Araki R., Iwanaga K., Ueda K., Isaka M. (2021). Intestinal Complication With Myxomatous Mitral Valve Diseases in Chihuahuas. Front. Vet. Sci..

[10] Prieto Ramos J., Corda A., Swift S., Saderi L., De La Fuente Oliver G., Corcoran B., Summers K.M., French A.T. (2021). Clinical and Echocardiographic Findings in an Aged Population of Cavalier King Charles Spaniels. Animals.

[11] Chetboul V., Tissier R. (2012). Echocardiographic assessment of canine degenerative mitral valve disease. J. Vet. Cardiol..

[12] Haggstrom J., Hansson K., Kvart C., Karlberg B.E., Vuolteenaho O., Olsson K. (1997). Effects of naturally acquired decompensated mitral valve regurgitation on the renin-angiotensin-aldosterone system and atrial natriuretic peptide concentration in dogs. Am. J. Vet. Res..

[13] Boswood A., Haggstrom J., Gordon S.G., Wess G., Stepien R.L., Oyama M.A., Keene B.W., Bonagura J., MacDonald K.A., Patteson M. (2016). Effect of Pimobendan in Dogs with Preclinical Myxomatous Mitral Valve Disease and Cardiomegaly: The EPIC Study-A Randomized Clinical Trial. J. Vet. Intern. Med..

[14] Lancellotti P., Tribouilloy C., Hagendorff A., Popescu B.A., Edvardsen T., Pierard L.A., Badano L., Zamorano J.L., Scientific Document Committee of the European Association of Cardiovascular Imaging (2013). Recommendations for the echocardiographic assessment of native valvular regurgitation: An executive summary from the European Association of Cardiovascular Imaging. Eur. Heart J. Cardiovasc. Imaging.

[15] Zoghbi W.A., Adams D., Bonow R.O., Enriquez-Sarano M., Foster E., Grayburn P.A., Hahn R.T., Han Y., Hung J., Lang R.M. (2017). Recommendations for Noninvasive Evaluation of Native Valvular Regurgitation: A Report from the American Society of Echocardiography Developed in Collaboration with the Society for Cardiovascular Magnetic Resonance. J. Am. Soc. Echocardiogr..

[16] Larouche-Lebel E., Loughran K.A., Oyama M.A. (2019). Echocardiographic indices and severity of mitral regurgitation in dogs with preclinical degenerative mitral valve disease. J. Vet. Intern. Med..

[17] Choi H., Lee K., Lee H., Lee Y., Chang D., Eom K., Youn H., Choi M., Yoon J. (2004). Quantification of mitral regurgitation using proximal isovelocity surface area method in dogs. J. Vet. Sci..

[18] Doiguchi O., Takahashi T. (2000). Examination of quantitative analysis and measurement of the regurgitation rate in mitral valve regurgitation by the “proximal isovelocity surface area” method. J. Vet. Med. Sci..

[19] Enriquez-Sarano M., Schaff H.V., Orszulak T.A., Tajik A.J., Bailey K.R., Frye R.L. (1995). Valve repair improves the outcome of surgery for mitral regurgitation. A multivariate analysis. Circulation.

[20] Gouni V., Serres F.J., Pouchelon J.L., Tissier R., Lefebvre H.P., Nicolle A.P., Sampedrano C.C., Chetboul V. (2007). Quantification of mitral valve regurgitation in dogs with degenerative mitral valve disease by use of the proximal isovelocity surface area method. J. Am. Vet. Med. Assoc..

[21] Kittleson M.D., Brown W.A. (2003). Regurgitant fraction measured by using the proximal isovelocity surface area method in dogs with chronic myxomatous mitral valve disease. J. Vet. Intern. Med..

[22] Schwammenthal E., Chen C., Giesler M., Sagie A., Guerrero J.L., Vazquez de Prada J.A., Hombach V., Weyman A.E., Levine R.A. (1996). New method for accurate calculation of regurgitant flow rate based on analysis of Doppler color flow maps of the proximal flow field. Validation in a canine model of mitral regurgitation with initial application in patients. J. Am. Coll. Cardiol..

[23] Baumgartner H., Hung J., Bermejo J., Chambers J.B., Edvardsen T., Goldstein S., Lancellotti P., LeFevre M., Miller F., Otto C.M. (2017). Recommendations on the Echocardiographic Assessment of Aortic Valve Stenosis: A Focused Update from the European Association of Cardiovascular Imaging and the American Society of Echocardiography. J. Am. Soc. Echocardiogr..

[24] Recusani F., Bargiggia G.S., Yoganathan A.P., Raisaro A., Valdes-Cruz L.M., Sung H.W., Bertucci C., Gallati M., Moises V.A., Simpson I.A. (1991). A new method for quantification of regurgitant flow rate using color Doppler flow imaging of the flow convergence region proximal to a discrete orifice. An in vitro study. Circulation.

[25] Berrezaie M., Connolly D., Cruzado J., Mederska E., Dukes-McEwan J., Humm K. (2023). Infective endocarditis in dogs in the UK: 77 cases (2009–2019). J. Small Anim. Pract..

[26] Muzzi R.A., de Araujo R.B., Muzzi L.A., Pena J.L., Silva E.F. (2003). Regurgitant jet area by Doppler color flow mapping: Quantitative assessment of mitral regurgitation severity in dogs. J. Vet. Cardiol..

[27] Tidholm A. (1997). Retrospective study of congenital heart defects in 151 dogs. J. Small Anim. Pract..

[28] Moya J.L., Darriba-Pollan J., Garcia-Lledo A., Taboada D., Catalan-Sanz P., Megias-Saez A., Guzman-Martinez G., Campuzano-Ruiz R., Asin-Cardiel E. (2006). [Evaluation of mitral regurgitation severity using a simplified method based on proximal flow convergence]. Rev. Esp. Cardiol..

[29] Pu M., Prior D.L., Fan X., Asher C.R., Vasquez C., Griffin B.P., Thomas J.D. (2001). Calculation of mitral regurgitant orifice area with use of a simplified proximal convergence method: Initial clinical application. J. Am. Soc. Echocardiogr..

[30] Tokushima T., Reid C.L., Gardin J.M. (2001). Left ventricular diastolic function in the elderly. Am. J. Geriatr. Cardiol..

[31] Fox P.R. (2012). Pathology of myxomatous mitral valve disease in the dog. J. Vet. Cardiol..

[32] Haggstrom J., Boswood A., O’Grady M., Jons O., Smith S., Swift S., Borgarelli M., Gavaghan B., Kresken J.G., Patteson M. (2013). Longitudinal analysis of quality of life, clinical, radiographic, echocardiographic, and laboratory variables in dogs with myxomatous mitral valve disease receiving pimobendan or benazepril: The QUEST study. J. Vet. Intern. Med..

[33] Uretsky S., Morales D.C.V., Aldaia L., Mediratta A., Koulogiannis K., Marcoff L., Sakul S., Wolff S.D., Gillam L.D. (2021). Characterization of Primary Mitral Regurgitation With Flail Leaflet and/or Wall-Impinging Flow. J. Am. Coll. Cardiol..

[34] Hollmer M., Willesen J.L., Tolver A., Koch J. (2017). Left atrial volume and function in dogs with naturally occurring myxomatous mitral valve disease. J. Vet. Cardiol..

[35] Thomas W.P., Gaber C.E., Jacobs G.J., Kaplan P.M., Lombard C.W., Moise N.S., Moses B.L. (1993). Recommendations for standards in transthoracic two-dimensional echocardiography in the dog and cat. Echocardiography Committee of the Specialty of Cardiology, American College of Veterinary Internal Medicine. J. Vet. Intern. Med..

[36] Hanton G., Geffray B., Lodola A. (1998). Echocardiography, a non-invasive method for the investigation of heart morphology and function in laboratory dogs: 1. Method and reference values for M-mode parameters. Lab. Anim..

[37] Cornell C.C., Kittleson M.D., Della Torre P., Haggstrom J., Lombard C.W., Pedersen H.D., Vollmar A., Wey A. (2004). Allometric scaling of M-mode cardiac measurements in normal adult dogs. J. Vet. Intern. Med..

[38] Hansson K., Haggstrom J., Kvart C., Lord P. (2002). Left atrial to aortic root indices using two-dimensional and M-mode echocardiography in cavalier King Charles spaniels with and without left atrial enlargement. Vet. Radiol. Ultrasound.

[39] Schiller N.B., Shah P.M., Crawford M., DeMaria A., Devereux R., Feigenbaum H., Gutgesell H., Reichek N., Sahn D., Schnittger I. (1989). Recommendations for quantitation of the left ventricle by two-dimensional echocardiography. American Society of Echocardiography Committee on Standards, Subcommittee on Quantitation of Two-Dimensional Echocardiograms. J. Am. Soc. Echocardiogr..

[40] Boon J.A. (2011). Veterinary Echocardiography.

[41] The R Project for Statistical Computing. https://www.r-project.org.

[42] Wuensch K.L., Evans J.D. (1996). Straightforward Statistics for the Behavioral Sciences. J. Am. Stat. Assoc..

[43] Nahm F.S. (2022). Receiver operating characteristic curve: Overview and practical use for clinicians. Korean J. Anesthesiol..

[44] Popovic Z.B., Thomas J.D. (2017). Assessing observer variability: A user’s guide. Cardiovasc. Diagn. Ther..

[45] Vignola P.A., Bloch A., Kaplan A.D., Walker H.J., Chiotellis P.N., Myers G.S. (1977). Interobserver variability in echocardiography. J. Clin. Ultrasound.

[46] Landis J.R., Koch G.G. (1977). The measurement of observer agreement for categorical data. Biometrics.

[47] Simpson I.A., Shiota T., Gharib M., Sahn D.J. (1996). Current status of flow convergence for clinical applications: Is it a leaning tower of “PISA”?. J. Am. Coll. Cardiol..

[48] Yosefy C., Levine R.A., Solis J., Vaturi M., Handschumacher M.D., Hung J. (2007). Proximal flow convergence region as assessed by real-time 3-dimensional echocardiography: Challenging the hemispheric assumption. J. Am. Soc. Echocardiogr..

[49] Topilsky Y., Michelena H., Bichara V., Maalouf J., Mahoney D.W., Enriquez-Sarano M. (2012). Mitral valve prolapse with mid-late systolic mitral regurgitation: Pitfalls of evaluation and clinical outcome compared with holosystolic regurgitation. Circulation.

[50] Borgarelli M., Crosara S., Lamb K., Savarino P., La Rosa G., Tarducci A., Haggstrom J. (2012). Survival characteristics and prognostic variables of dogs with preclinical chronic degenerative mitral valve disease attributable to myxomatous degeneration. J. Vet. Intern. Med..

[51] Lisciandro G.R., Fosgate G.T., Fulton R.M. (2014). Frequency and number of ultrasound lung rockets (B-lines) using a regionally based lung ultrasound examination named vet BLUE (veterinary bedside lung ultrasound exam) in dogs with radiographically normal lung findings. Vet. Radiol. Ultrasound.

[52] Vezzosi T., Mannucci T., Pistoresi A., Toma F., Tognetti R., Zini E., Domenech O., Auriemma E., Citi S. (2017). Assessment of Lung Ultrasound B-Lines in Dogs with Different Stages of Chronic Valvular Heart Disease. J. Vet. Intern. Med..

[53] Hagendorff A., Knebel F., Helfen A., Stobe S., Haghi D., Ruf T., Lavall D., Knierim J., Altiok E., Brandt R. (2021). Echocardiographic assessment of mitral regurgitation: Discussion of practical and methodologic aspects of severity quantification to improve diagnostic conclusiveness. Clin. Res. Cardiol..

[54] Zoghbi W.A., Enriquez-Sarano M., Foster E., Grayburn P.A., Kraft C.D., Levine R.A., Nihoyannopoulos P., Otto C.M., Quinones M.A., Rakowski H. (2003). Recommendations for evaluation of the severity of native valvular regurgitation with two-dimensional and Doppler echocardiography. J. Am. Soc. Echocardiogr..

